# The tip of the iceberg: lipomatous tumours presenting as abdominal or pelvic wall hernias

**DOI:** 10.1186/s13244-019-0739-1

**Published:** 2019-07-05

**Authors:** Kirsten van Langevelde, Christine Azzopardi, Gareth Kiernan, Max Gibbons, Zsolt Orosz, James Teh

**Affiliations:** 1 Department of Radiology, Nuffield Orthopaedic Centre, Windmill Road, Headington, Oxford, OX3 7HE UK; 2 Department of Orthopaedics, Rheumatology and Musculoskeletal Sciences, Nuffield Orthopaedic Centre, Windmill Road, Headington, Oxford, OX3 7HE UK; 3 Department of Histopathology, Nuffield Orthopaedic Centre, Windmill Road, Headington, Oxford, OX3 7HE UK

**Keywords:** Hernia, Lipomatous tumour, Soft tissue tumour, MRI, Abdominal wall, Pelvis

## Abstract

Liposarcomas are the most common soft tissue sarcoma. They occur mainly in the thigh or retroperitoneum. Due to their size, lipomatous tumours can herniate either through the abdominal wall or in the groin. The part of the tumour that herniates represents only the ‘tip of the iceberg’, as the main part of the tumour is not detectable clinically and is often underestimated. Due to their deep location, lipomatous tumours are often large at the time of presentation and therefore their surgical management can be challenging. Furthermore, due to their delayed presentation, there is a higher risk of de-differentiation. In this pictorial review, we discuss different presentations of herniating lipomatous tumours according to the location of the abdominal wall defects. We aim to cover a wide spectrum of hernia defects including inguinal, ventral, lumbar, sciatic and ischiorectal hernias. We also present cases of tumours within the psoas compartment ‘herniating’ from the pelvis into the thigh. In case of a palpable lump, the first diagnostic step is to perform an ultrasound. If the herniating tissue is not fully accessible with ultrasound, additional cross-sectional imaging by CT or MRI is warranted. In this article, CT and MRI findings in lipomatous tumours are addressed and the use of contrast enhanced sequences in MRI is discussed. Patients’ outcome depends not only on adequate diagnosis but also on the correct route of tissue sampling for histology and oncological resection to prevent local recurrence and loss of function. Therefore, referral to a specialised sarcoma treatment centre is key and needs to be done before biopsy.

## Key points

- Deep lipomatous tumours are often large but may present with a small herniating palpable mass
- The herniating part of the tumour represents the ‘tip of the iceberg’, as the main part of the tumour is not detectable clinically and is underestimated
- General ultrasound assessment of an inguinal hernia can be the moment of detecting a lipomatous tumour
- If the herniating tissue is not fully accessible with ultrasound, additional cross-sectional imaging by CT or MRI is warranted
- Referral to a specialised sarcoma treatment centre is needed before biopsy and resection, to avoid traversing uninvolved compartments and needle tract seeding

## Introduction

Liposarcomas are the most common soft tissue sarcomas which occur predominantly in the thigh or retroperitoneum. Due to their size, lipomatous tumours can present as hernias either in the abdominal wall or in the groin. The part of the tumour that herniates represents only the ‘tip of the iceberg’, as the main part of the tumour is not detectable clinically and is often underestimated. Due to their deep location, lipomatous tumours are often large at the time of presentation and therefore their surgical management can be challenging. Secondly, with late presentation, there is a higher risk of de-differentiation [1].

**Fig. 1:**
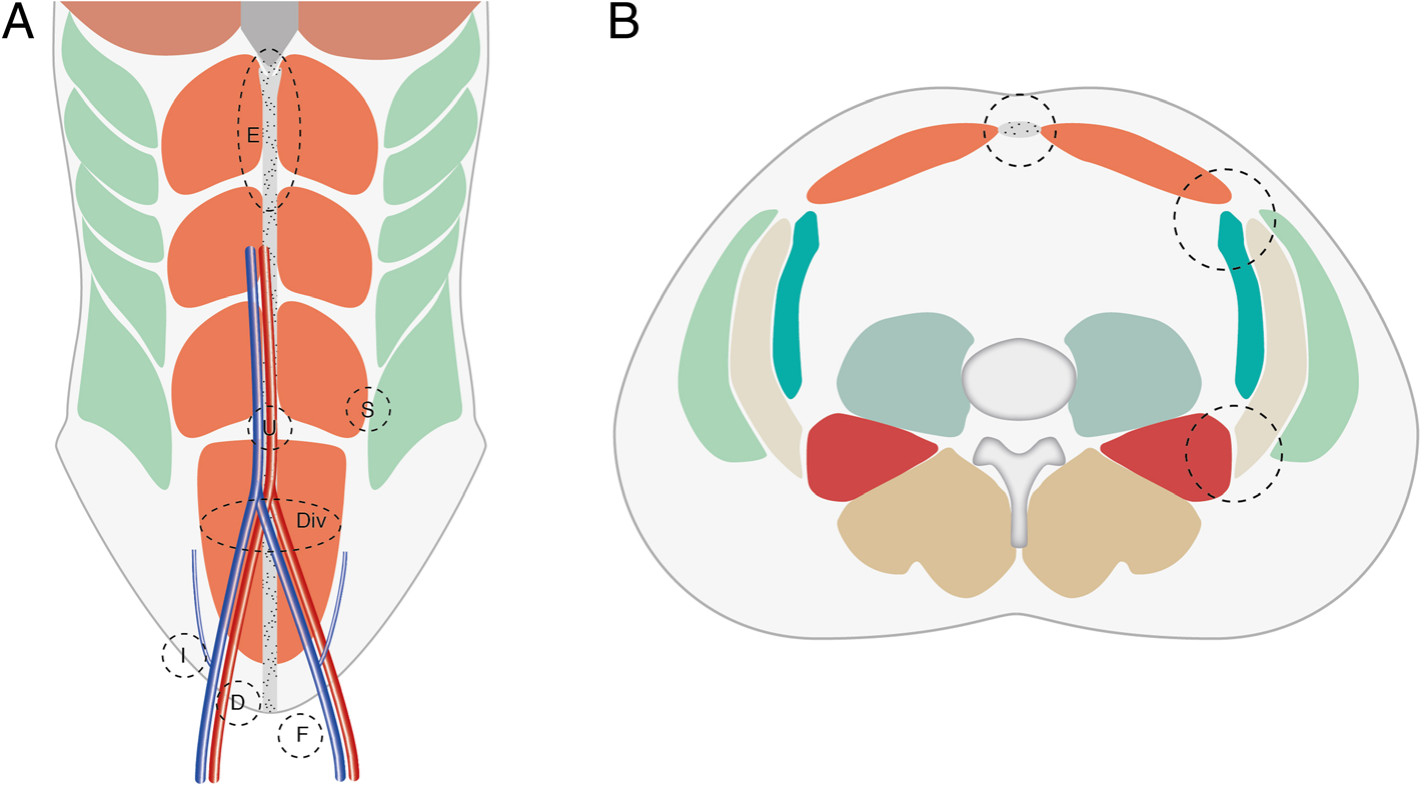
**a** Schematic coronal drawing of the anterior abdominal wall muscles with the typical hernia locations marked by circles with dotted lines. Abbreviations stand for: *E* epigastric, *S* Spigelian hernia (between rectus and oblique abdominal muscles), *Div* rectus divarication, *I* indirect inguinal hernia (lateral to the origin of the epigastric vessels), *D* direct inguinal hernia (medial to the epigastric vessels), *F* femoral (inferior to the inguinal ligament and medial to the femoral vein), *U* umbilical. **b** Schematic axial drawing of the abdomen with hernia locations marked by the circles with dotted lines. Anteriorly, in the midline there is the umbilical or paraumbilical hernia (small circle). Between the recti (orange) and the transverse (dark green), internal oblique (beige) and external oblique (light green) abdominal muscles there is the Spigelian hernia and the interparietal wall hernia also occurs here. Posterolaterally, the lumbar hernia arises between the internal oblique abdominal muscle and the quadratus lumborum muscle (red)

In this pictorial review, we discuss different presentations of herniating lipomatous tumours according to the location of the abdominal wall defects (for a schematic representation see Fig. 1a, b). We aim to cover a wide spectrum of hernia defects including inguinal, ventral, lumbar, sciatic and ischiorectal hernias. We also present cases of tumours within the psoas compartment ‘herniating’ from the pelvis into the thigh, although these are not strictly true hernias.

The World Health Organisation classification of soft tissue and bone tumours recognises four subtypes of liposarcomas: the atypical lipomatous tumour (ALT) or well-differentiated lipomatous tumour, the de-differentiated liposarcoma, the myxoid liposarcoma and the pleomorphic liposarcoma [1, 2].

Cytogenetic analysis, i.e. MDM2 and CDK4 amplification, is key for histological differentiation of a benign lipomatous lesion from an ALT. Up to one-third of ALTs recur locally; however, metastatic spread is only rarely seen: at the time when de-differentiation occurs. The most important prognostic factor is anatomical location [1].

Computed tomography (CT) and magnetic resonance (MR) imaging are the primary modalities used in evaluating abdominal and pelvic lesions. Cross-sectional imaging not only facilitates diagnosis of the primary lesion but also delineates the degree of local and metastatic invasion and enables pre-operative surgical planning.

In this article, we briefly review the different types of hernias, followed by case presentations. All cases presented to the radiology department at the Nuffield Orthopaedic Centre and were discussed in the MDT sarcoma meeting in the last 3 years. Finally, a practical approach for radiological interpretation of these herniating soft tissue tumours and their concerning features on imaging will be addressed in the discussion.

## Types of hernias

### Direct inguinal hernia

Direct inguinal hernias are often secondary to weakness of the transversalis fascia. They are located medial to the inferior epigastric vessels and occur more in men than in women, often bilaterally [3, 4].

### Indirect inguinal hernia

Indirect inguinal hernias are the most common type of abdominal wall hernias. These hernias are located lateral to the inferior epigastric vessels and result from a patent processus vaginalis. This results in herniation along the spermatic cord into the scrotum in men whilst in women this follows the round ligament into the labia majora [3].

### Spigelian hernia

The linea semilunaris is a fibrous union of the rectus sheath with the aponeuroses of the transverse and oblique abdominal muscles extending from the level of the ninth costal cartilage to the symphysis pubis. A Spigelian hernia is the result of a defect in the linea semilunaris [4].

### Rectus divarication

Also known as separation of the rectus abdominis muscles (along the length of the linea alba), this entity occurs most commonly in elderly multiparous women.

### Lumbar hernia

Lumbar hernias occur through defects in the lumbar muscles or the posterior transversalis or lumbodorsal fascia. The diffuse type of lumbar hernia is often iatrogenic and may occur following surgical incisions [4].

### Sciatic hernia

Sciatic hernias are rare and can occur in the greater or lesser sciatic foramina, the majority are found in elderly women. About one-third of patients present with a reducible gluteal mass; other symptoms are non-specific abdominal or pelvic pain. CT can result in a false negative scan, as this is performed with the patient in supine position potentially compressing the lesion. If the CT is negative in the presence of a high clinical suspicion, ultrasound has been proposed as an alternative modality as it adds a dynamic component to the assessment with the Valsalva manoeuvre and the patient in standing position. Magnetic resonance imaging (MRI) is the modality of choice to evaluate the sciatic nerve in cases of sciatic hernia [5, 6].

### Iliopsoas compartment ‘hernia’

The iliopsoas compartment is composed of the psoas major, psoas minor (present in 40% of people) and iliacus muscles which have a common fascial sheath and fuse to form a common tendinous insertion onto the lesser trochanter. Violation of the retroperitoneal fascial planes by neoplastic processes may give rise to lesions within this compartment and the iliopsoas compartment may act as a conduit for the distant spread of disease. Sarcomas in the iliopsoas compartment have a poorer outcome than those located in other areas, as the tumour can grow undetected for longer periods of time. The tumours in this compartment are often surgically inaccessible due to their proximity to vital structures [7, 8].

### Ischiorectal hernia

An ischiorectal hernia extends from the pelvis through a gap between the levator ani muscle and the coccygeal muscle, which is covered only by fascia. A tumour arising in the pelvis can extend into the perineal fat through this gap by invading the muscles. The so-called ‘pelvic outlet’ is a route of tumour extension between the pelvic cavity and the perineum [9].

## Case presentations

### Indirect inguinal hernias

#### Case 1A

**Fig. 2:**
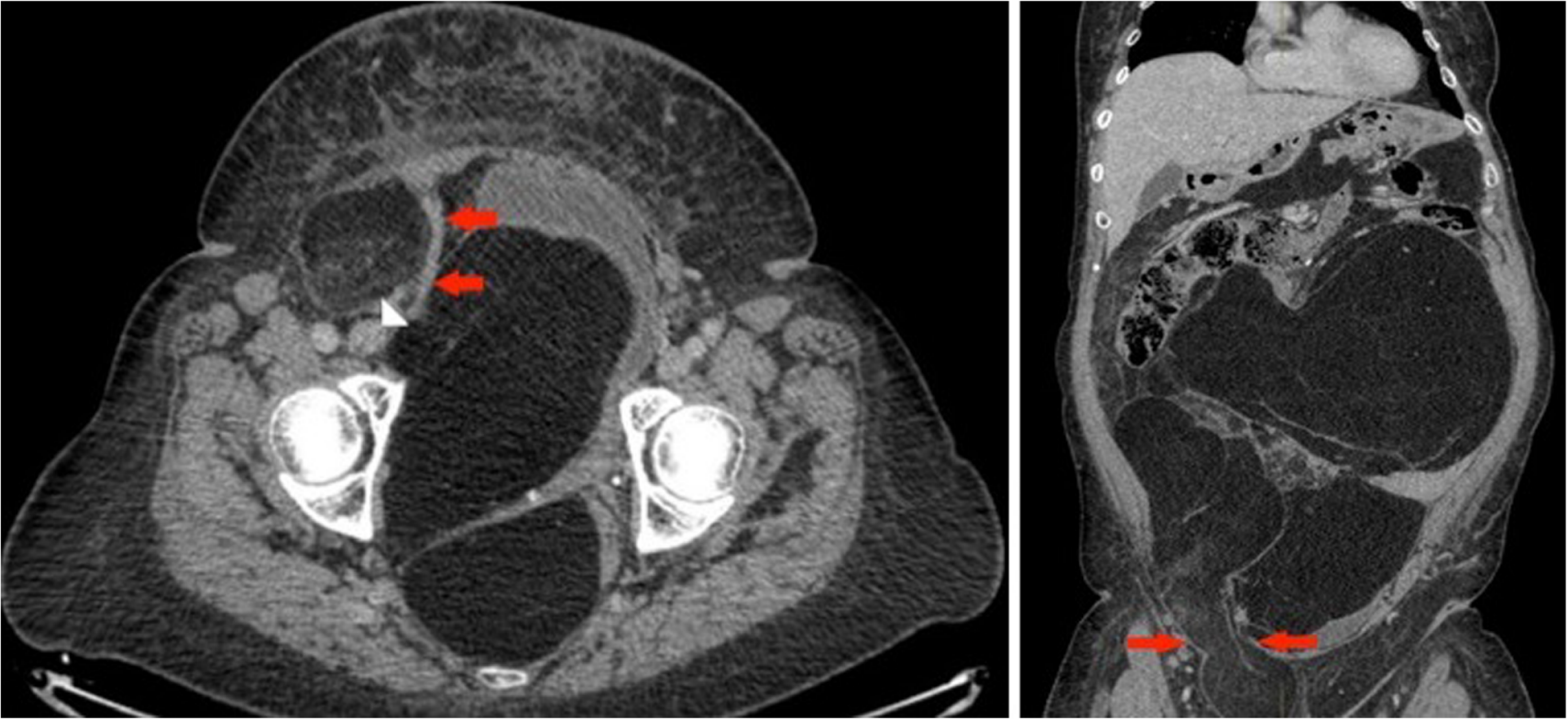
Left panel: Axial non-contrast-enhanced CT: a large pelvic mass predominantly of fat density is seen to compress and displace the bladder anteriorly. Part of the mass also extends into the right groin lateral to the epigastric vessels which are stretched over the lesion medially (red arrows). The white arrowhead points towards the origin of the inferior epigastric vessels from the external iliac vessels. Right panel: Coronal reconstruction of a non-contrast-enhanced CT, demonstrating the hernial neck (red arrows) as the tumour extends into the right inguinal canal

A 63-year-old woman presented with abdominal distension and increased weight, after having a lipomatous tumour being resected 10 years ago. CT shows the inferior epigastric vessels which are stretched over the tumour. Histopathology confirmed recurrence of a well-differentiated liposarcoma (Fig. 2).

#### Case 1B

**Fig. 3:**
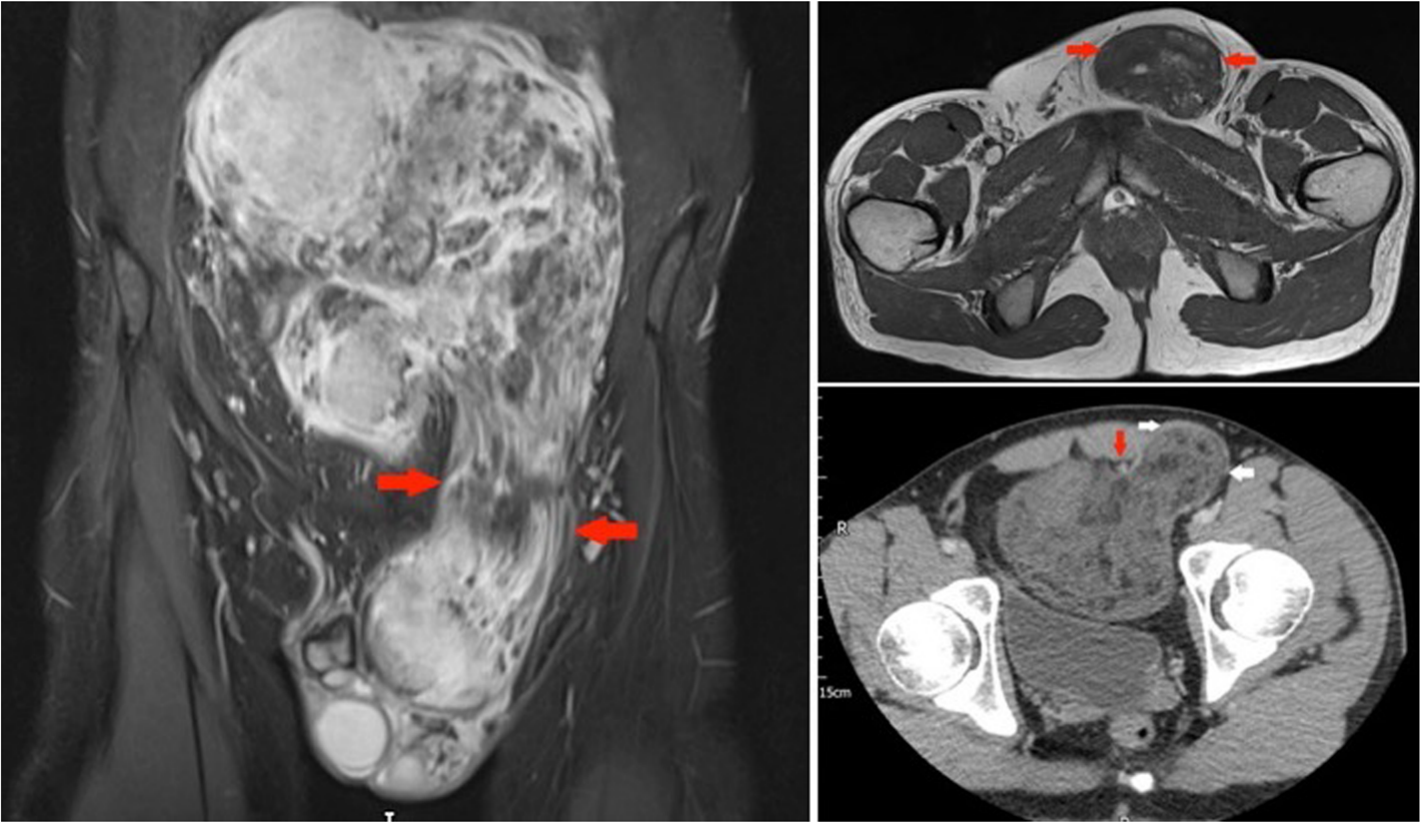
Left panel: Coronal T1 TIRM-weighted MR image of the abdomen and pelvis: large intra-abdominal mass with areas of fat suppression suggesting a fat containing tumour. The tumour is seen to herniate through the left inguinal canal (red arrows). Right upper panel: Axial T1-weighted MR image of the pelvis: A large left indirect inguinal hernia is observed (red arrows). On T1-weighted imaging fat content is identified which would suggest liposarcoma. Right lower panel: Axial contrast-enhanced CT of the abdomen and pelvis: a large intra-pelvic lesion is observed herniating through a groin defect (white arrows) lateral to the inferior epigastric vessels (red arrow). It appears to be displacing and compressing the bladder posteriorly

A 34-year-old male presented with a rapidly enlarging abdominal mass which extended into his left groin. Although the swelling had been present for over a year, it had doubled in size in a few months’ time (Fig. 3).

CT and MR imaging confirmed areas of fat within the tumour on both modalities. On positron emission tomography computed tomography (PET CT), the tumour demonstrated a standardised uptake value (SUV) max of 3.8. No metastases were present at the time of imaging.

Patient was referred to a tertiary referral centre for soft tissue sarcomas, where biopsy and surgery were performed. Subsequently, the histopathology of the tumour proved to be a mammary type myofibroblastoma. This is a very rare benign tumour.

### Direct inguinal hernia

#### Case 2

**Fig. 4:**
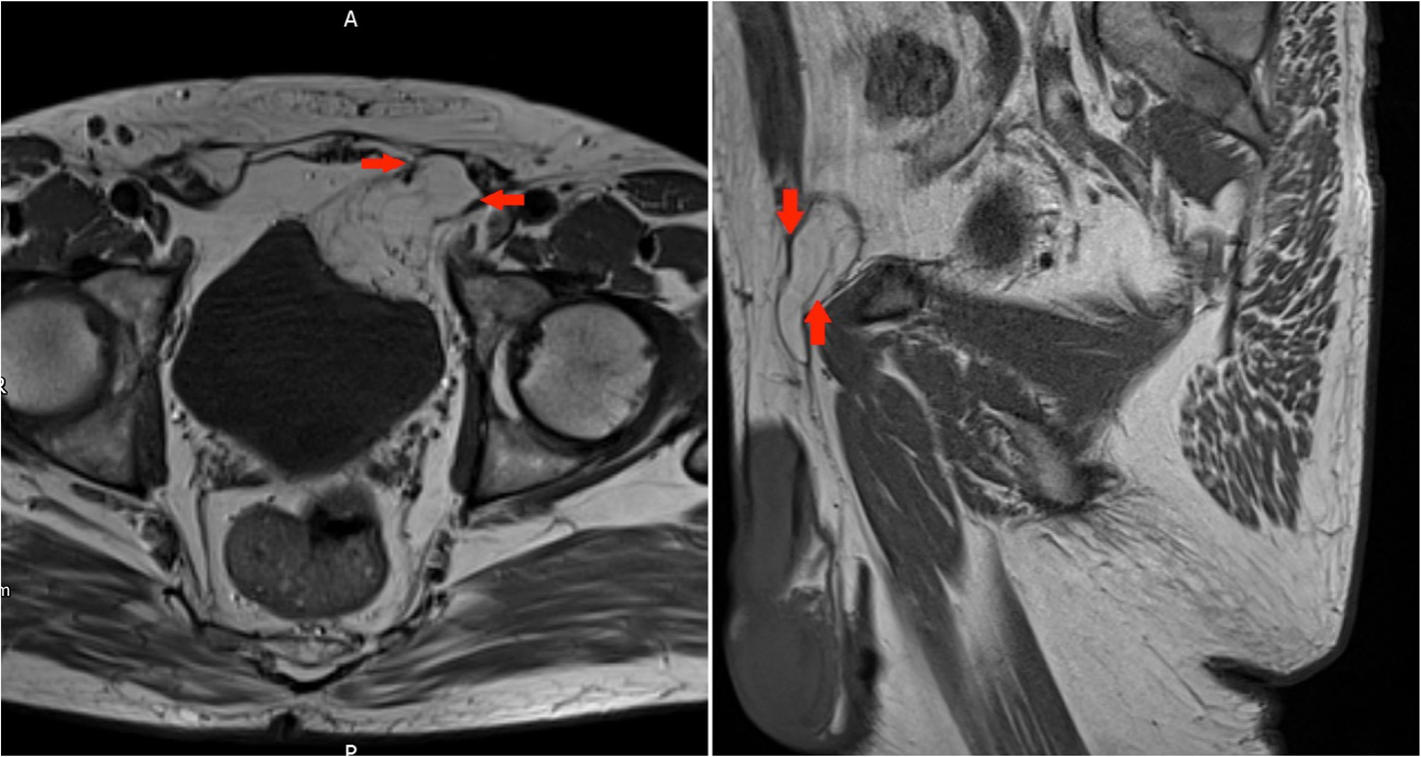
Left panel: Axial T1-weighted MR image of the pelvis: a lipomatous lesion arising within the pelvis and indenting the anterior bladder wall is seen with herniation (red arrows) medial to the epigastric vessels in keeping with a direct inguinal hernia. Right panel: Sagittal T1-weighted MR image of the pelvis: red arrows outline the neck of the direct inguinal hernia containing a lipomatous lesion. The hernial sac content is bright on T1 in keeping with fat, the presence of internal septations and ‘haziness’ of the fat content suggest pathology other than a simple lipomatous lesion

A 56-year-old male was referred for evaluation of an intra-pelvic lipomatous tumour discovered incidentally on CT colonoscopy, performed for a recent weight loss of 10 kg. Unfortunately, no histology was available for this patient; however, radiological features are in keeping with a lipomatous lesion. Open mesh repair of the hernia was performed (Fig. 4).

### Epigastric hernia

#### Case 3

**Fig. 5:**
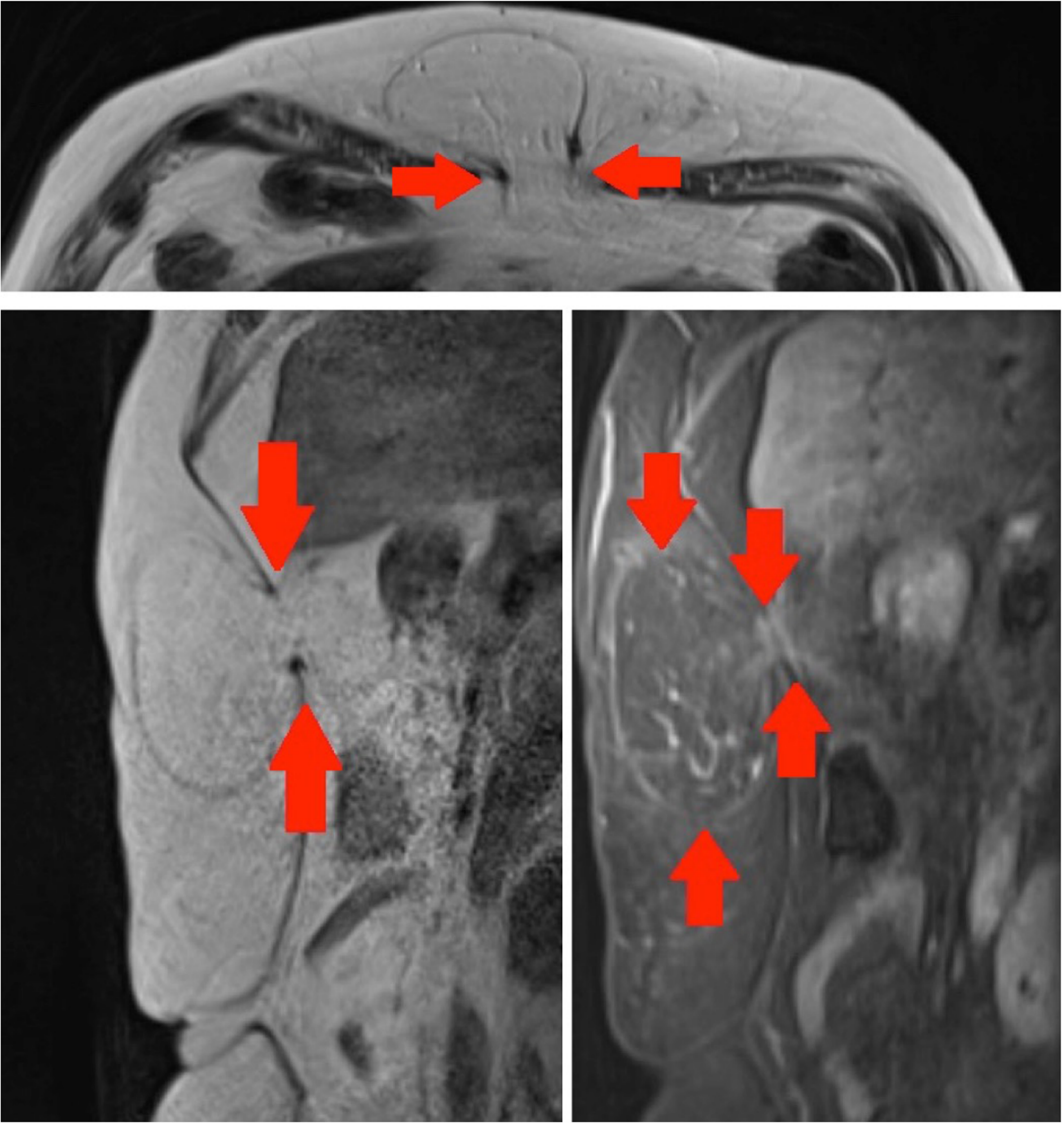
Top panel: Axial T1-weighted image of the upper abdomen: arrows delineate the defect in the anterior abdominal wall. The hernia sac contains fat with internal striations. Bottom left panel: Sagittal T1-weighted image of the upper abdomen: demonstrates an epigastric hernia with the hernial neck outlined by the red arrows. The hernial sac contents are of fat signal intensity and histology proved to be in keeping with ALT. Bottom right panel: Sagittal T1 TIRM-weighted image of the upper abdomen: Red arrows outline the epigastric hernia which demonstrates suppression on fat saturated sequences confirming the presence of a fat containing tumour. The presence of high signal septae suggests an atypical lipomatous tumour radiologically

A 60-year-old female presented with an enlarging lump in the epigastrium, which had doubled in size over a period of 6 months. Consequently, she was referred to our centre by her general practitioner (GP). Morphologic histopathology findings were in keeping with an ALT (demonstrated by oedema and fibrosis present within the lesion) (Fig. 5).

### Spigelian hernia

#### Case 4

**Fig. 6:**
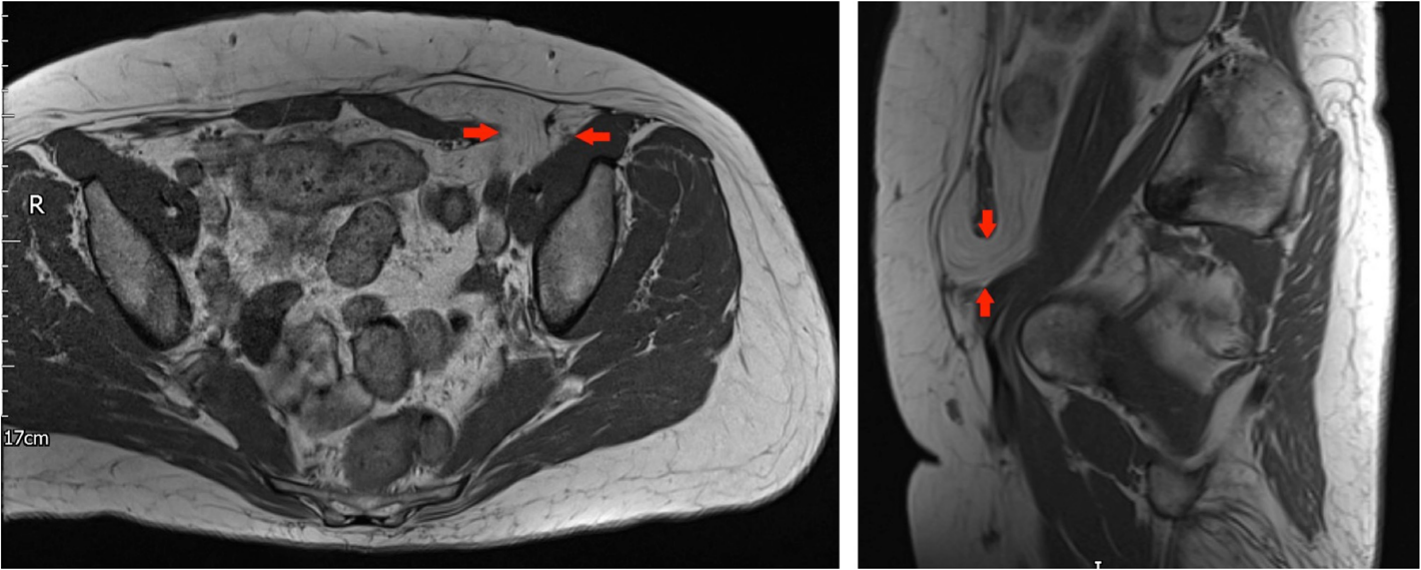
Left panel: Axial T1-weighted MRI of the pelvis and lower abdomen: red arrows outline a defect in the linea semilunaris with a fat containing hernial sac. Right panel: Sagittal T1-weighted MRI of the pelvis and lower abdomen: Spigelian hernia outlined by red arrows with hernial sac extending to the level of the pubic symphysis

A 61-year-old lady presented with weight loss and abdominal pain. Cross-sectional imaging revealed a fatty mass in the left lower quadrant extending caudally to the level of the pubic symphysis, with internal septations and haziness within the fat, suggestive of an ALT radiologically. Histopathology findings did not confirm atypia or signs of malignancy (Fig. 6).

### Divarication of the recti

#### Case 5

**Fig. 7:**
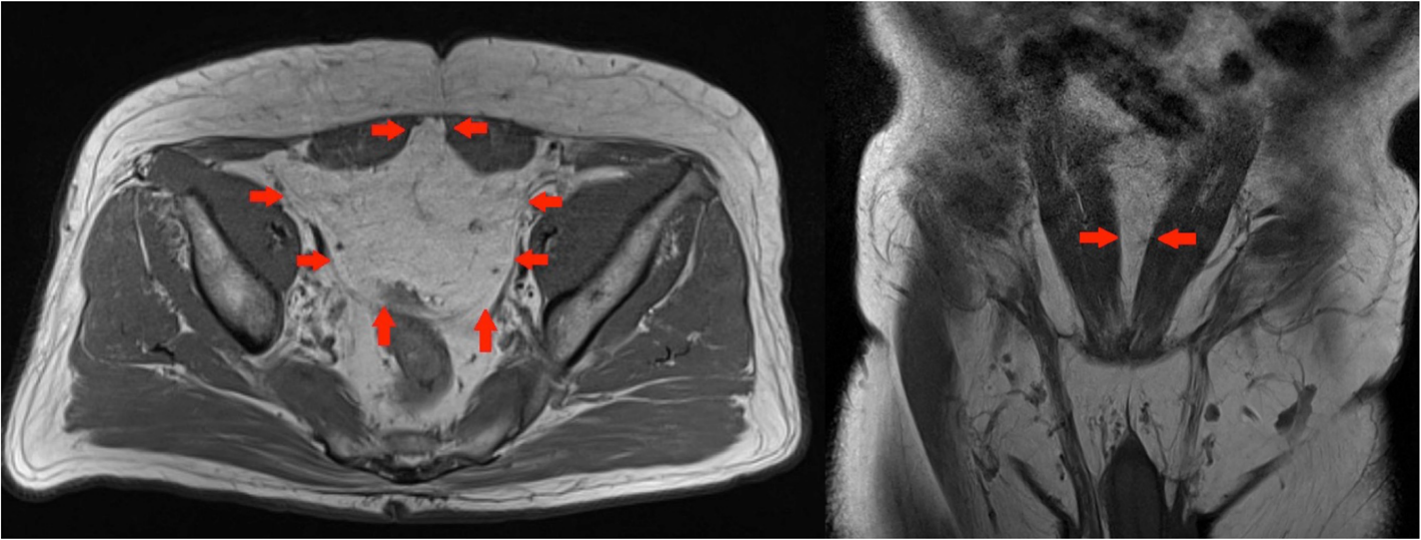
Left panel: Axial T1-weighted MRI of the pelvis and lower abdomen: red arrows outline the intra-abdominal lipomatous lesion with high T1 signal. Anteriorly the lesion is seen to split the recti in keeping with divarication. Right panel: Coronal T1-weighted MRI of the pelvis and lower abdomen: divarication of the recti (red arrows) secondary to a fat containing lesion in the abdomen

A 63-year-old male presented with recurrent burping and dysphagia without an obvious cause. As part of his work up, a CT of the abdomen was performed which picked up a large intra-abdominal lipomatous lesion as an incidental finding. Excision was performed and histopathology findings were in keeping with a benign lipoma. No MDM2 or CDK4 amplifications were present (Fig. 7).

### Lumbar hernias

#### Case 6A

**Fig. 8:**
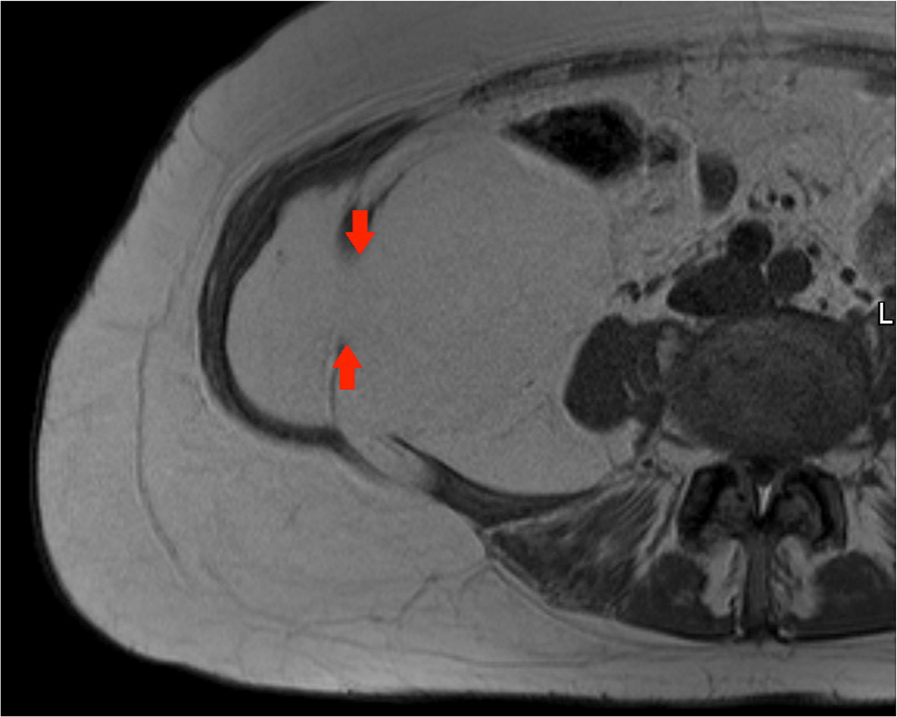
Axial T1-weighted MRI of the pelvis and lower abdomen: Large right sided lipomatous tumour extends laterally splitting the transversalis fascia and extends into the fascial planes of the obliques

A 71-year-old female presented with right sided colicky abdominal pain. She was worked up for biliary colic and a large lipomatous lesion measuring up to 19 cm was identified on cross-sectional imaging. Its size and radiological appearance suggested an atypical lipomatous lesion. On cytogenetic analysis, there was no MDM2 or CDK4 amplification (Fig. 8).

#### Case 6B

**Fig. 9:**
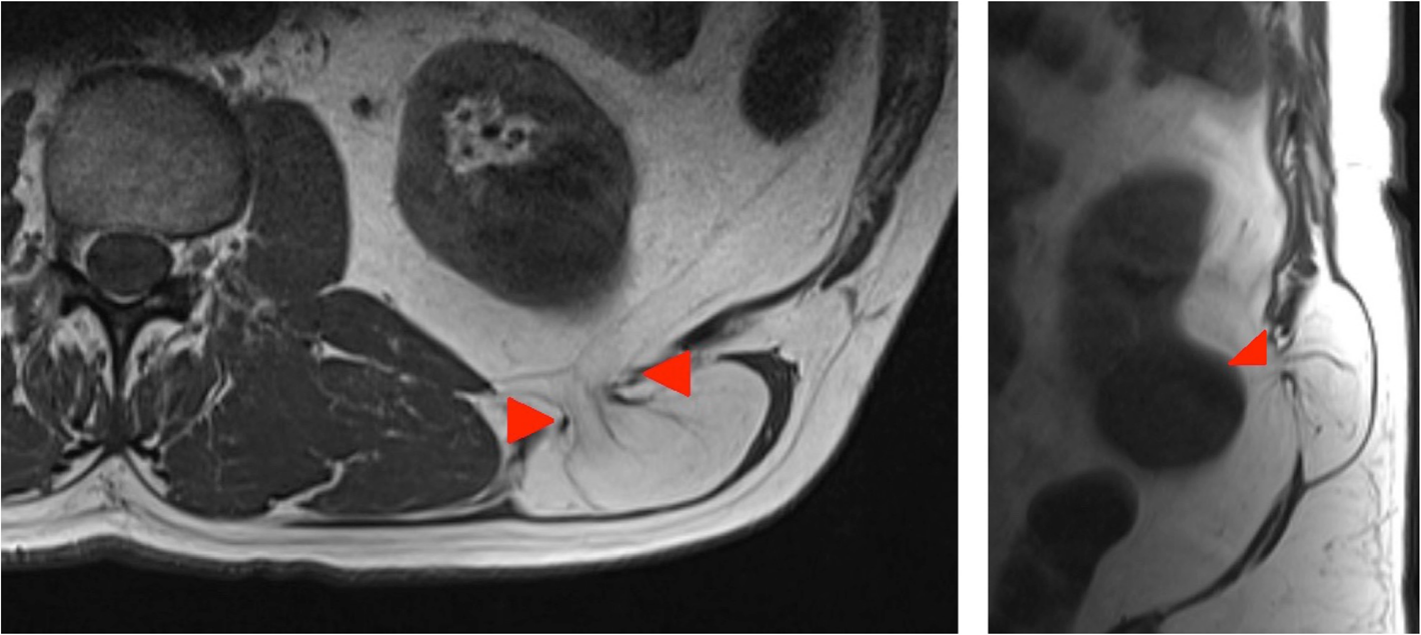
Left panel: Axial T1-weighted MR image demonstrates a defect in the left abdominal wall at the level of the inferior lumbar triangle, with protrusion of a retroperitoneal tumour with predominant fat signal intensity into the subcutaneous fat (red arrows). Right panel: Sagittal T1-weighted image: red arrowhead points out the hernial defect in the inferior lumbar triangle

A 63-year-old gentleman presented with a long-standing swelling on the posterior chest wall which had become slightly tender recently. An MRI was performed which revealed a left lumbar hernia containing a fat predominant retroperitoneal mass as an incidental finding (Fig. 9).

#### Case 6C

**Fig. 10:**
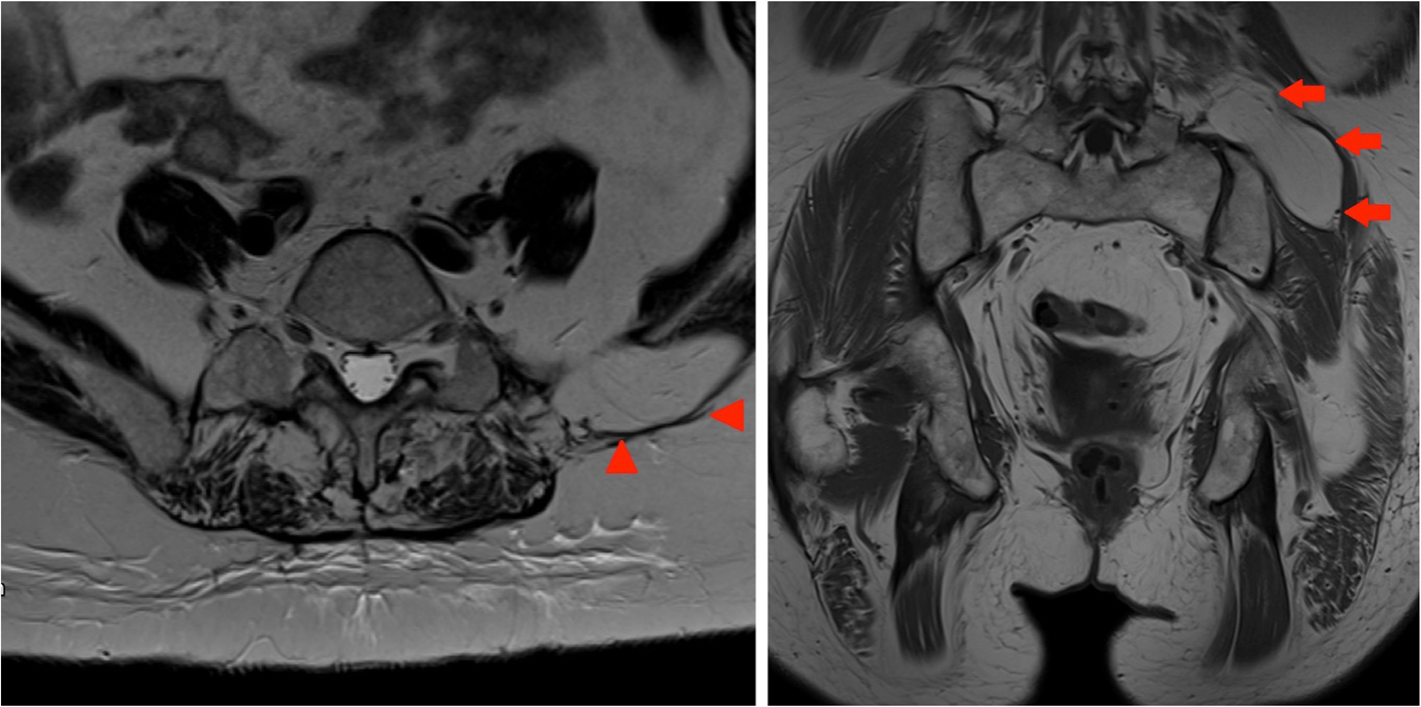
Left panel: Axial T2-weighted image of the pelvis: a fatty mass is seen to extend into the lumbar soft tissues with an intramuscular component in the gluteus medius. Right panel: Coronal T1-weighted image of the pelvis: red arrows outline the lumbar defect with fatty hernia contents

A 79-year-old lady complained of a long-standing discomfort in the lower back on the left. An MRI was performed for unrelated reasons as she also had a past medical history of colon cancer (Fig. 10).

### Interparietal hernia

#### Case 7

**Fig. 11:**
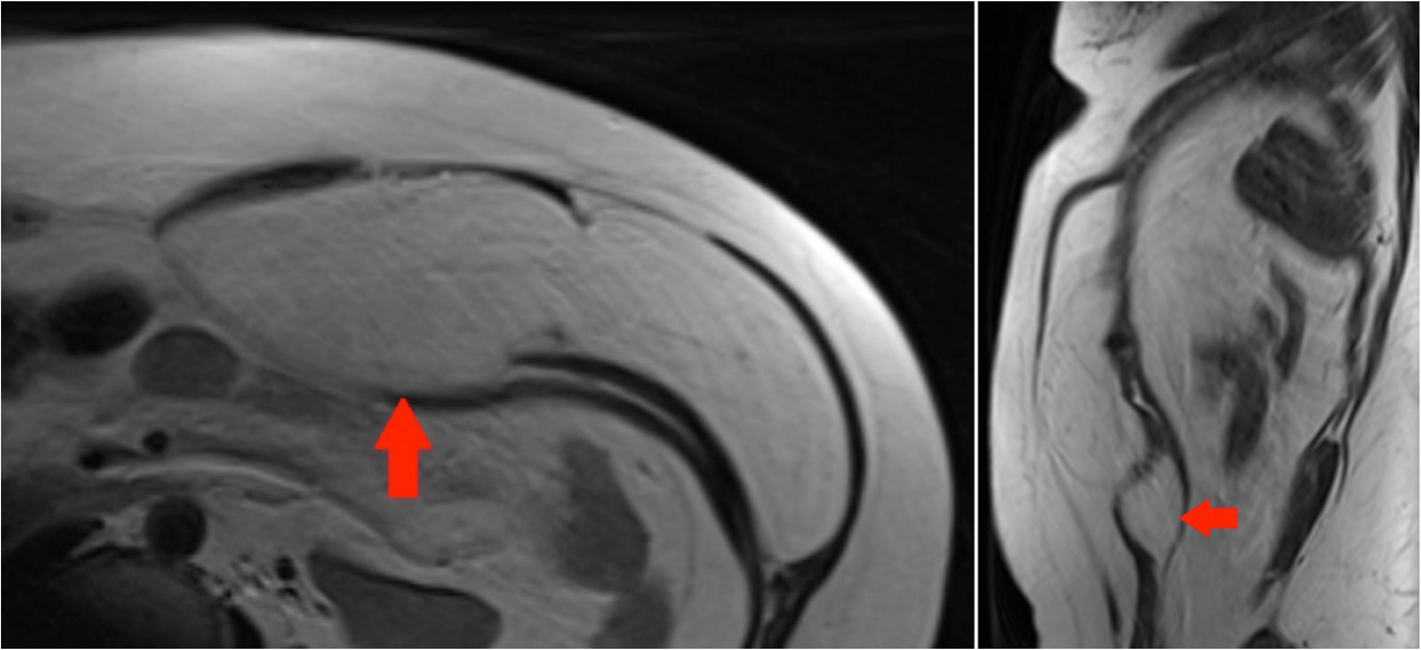
Left panel: Axial T1-weighted image of the abdomen: fatty lesion deep to the left rectus (red arrow) extends laterally under the external oblique muscle. Right panel: Sagittal T1-weighted MR image of the abdomen: interparietal hernia splitting the lateral abdominal wall muscles (red arrow)

A 53-year-old gentleman complained of a swelling over the left flank which appeared to be growing slowly over the period of a year. MRI confirmed the swelling to be an interparietal hernia secondary to a fatty tumour. Histology revealed this to be a benign lipomatous tumour (Fig. 11).

### Sciatic hernias

#### Case 8

A 58-year-old woman who had an incidental finding of a lipomatous mass on an MRI of the rectum which was performed for unrelated reasons, presented to our clinic with newly developed right-sided sciatica. Clinically, she had a palpable fullness in the right gluteal region.

**Fig. 12:**
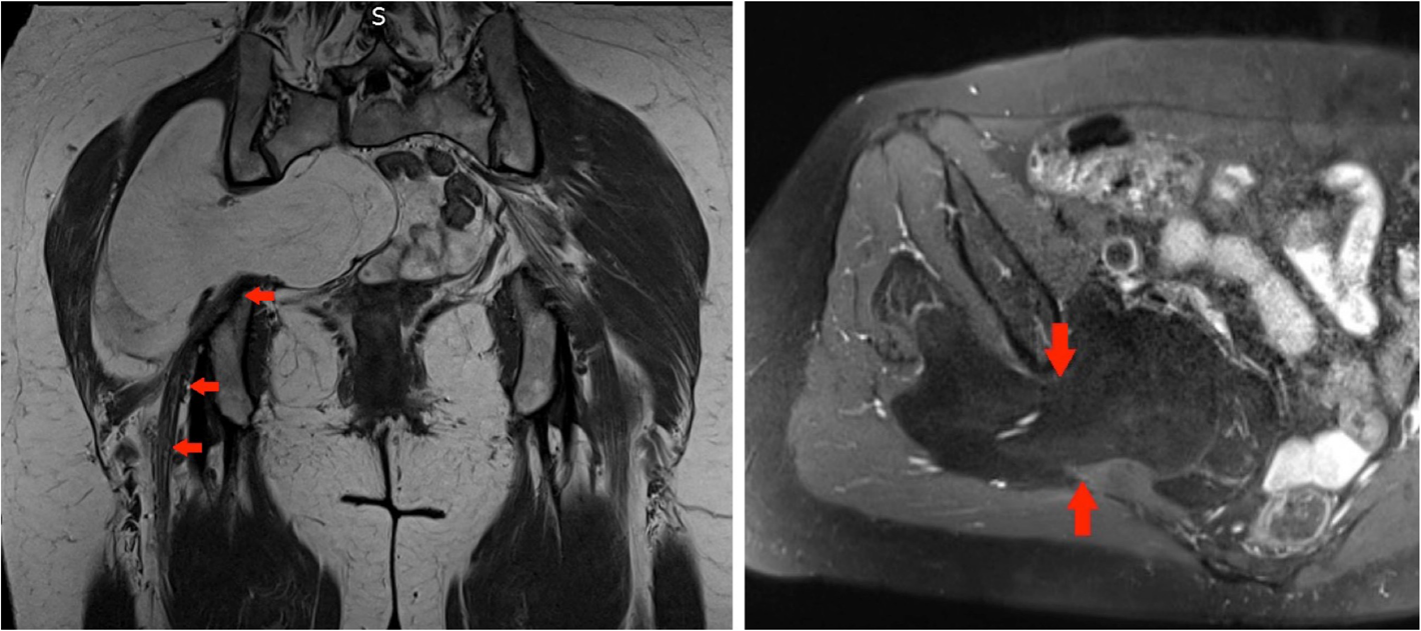
Left panel: Coronal T1-weighted MR image of the pelvis shows displacement and kinking of the right sciatic nerve at the level of the posterior acetabulum (red arrows). Right panel: Axial proton density fat sat weighted image of the pelvis confirms the lesion is predominantly fat containing as it supresses on fat saturated sequences. High signal septations which do not supress can also be visualised. Red arrows outline the greater sciatic notch

On MRI, a herniating fatty tumour is seen extending through the greater and lesser sciatic foramina (Fig. 12).

### Miscellaneous: ischiorectal fossa and iliopsoas compartment ‘hernias’

#### Case 9

**Fig. 13:**
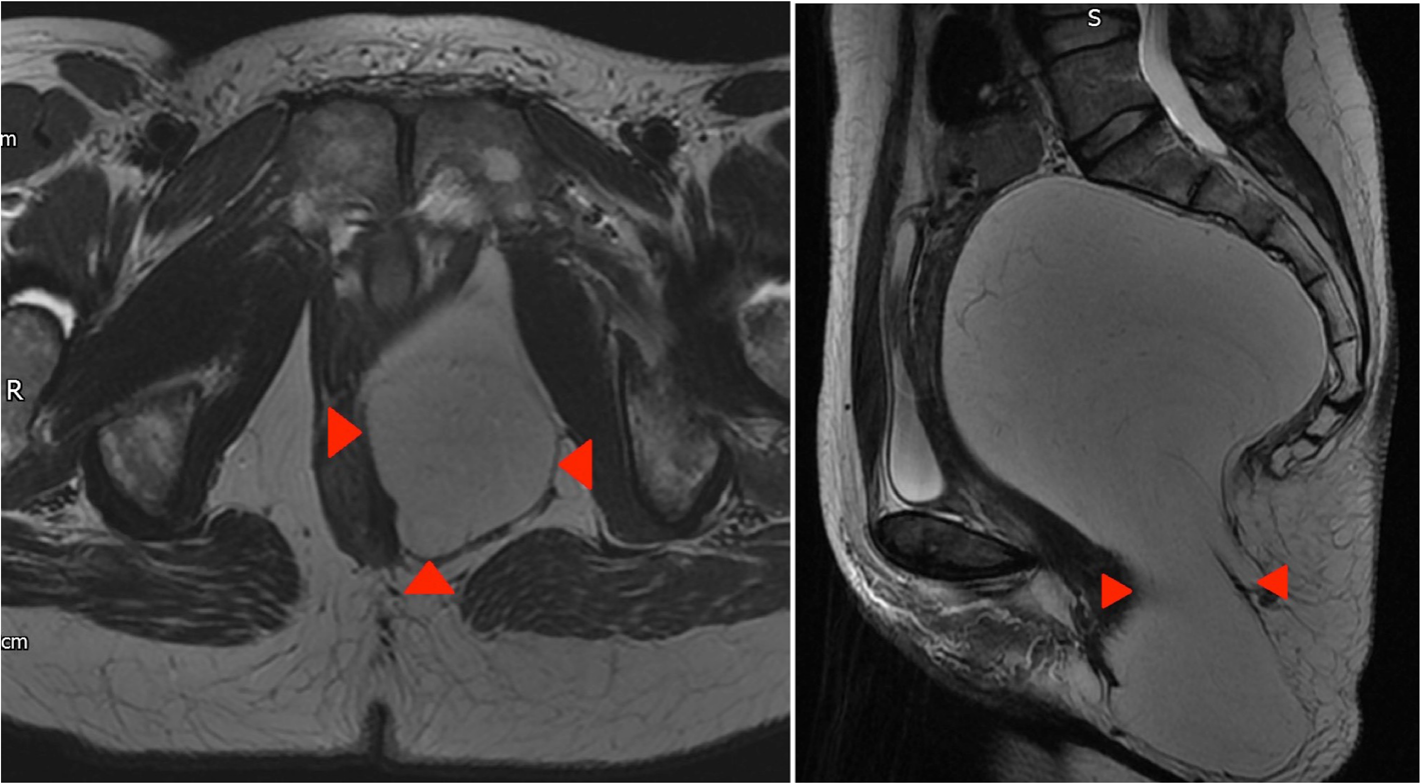
Left panel: Axial T2-weighted MR of the pelvis: arrowheads outline a fatty mass in the left ischiorectal fossa. Right panel: Sagittal T1-weighted MR image of the pelvis: large fatty pelvic lesion seen with extension through the left ischiorectal fossa into the perineal tissues. Arrowheads outline the level of the ischiorectal compartment

A 38-year-old lady presented to her GP with a history of intermenstrual bleeding. A pelvic ultrasound performed as part of the workup showed a mass, suspicious for an ovarian tumour. A pelvic MRI was then performed which revealed a large left ischiorectal fossa mass which extended into the perineum. Excision and histological examination followed which confirmed the presence of a lipomatous lesion with no MDM2 or CDK4 amplification (Fig. 13).

#### Case 10 A

**Fig. 14:**
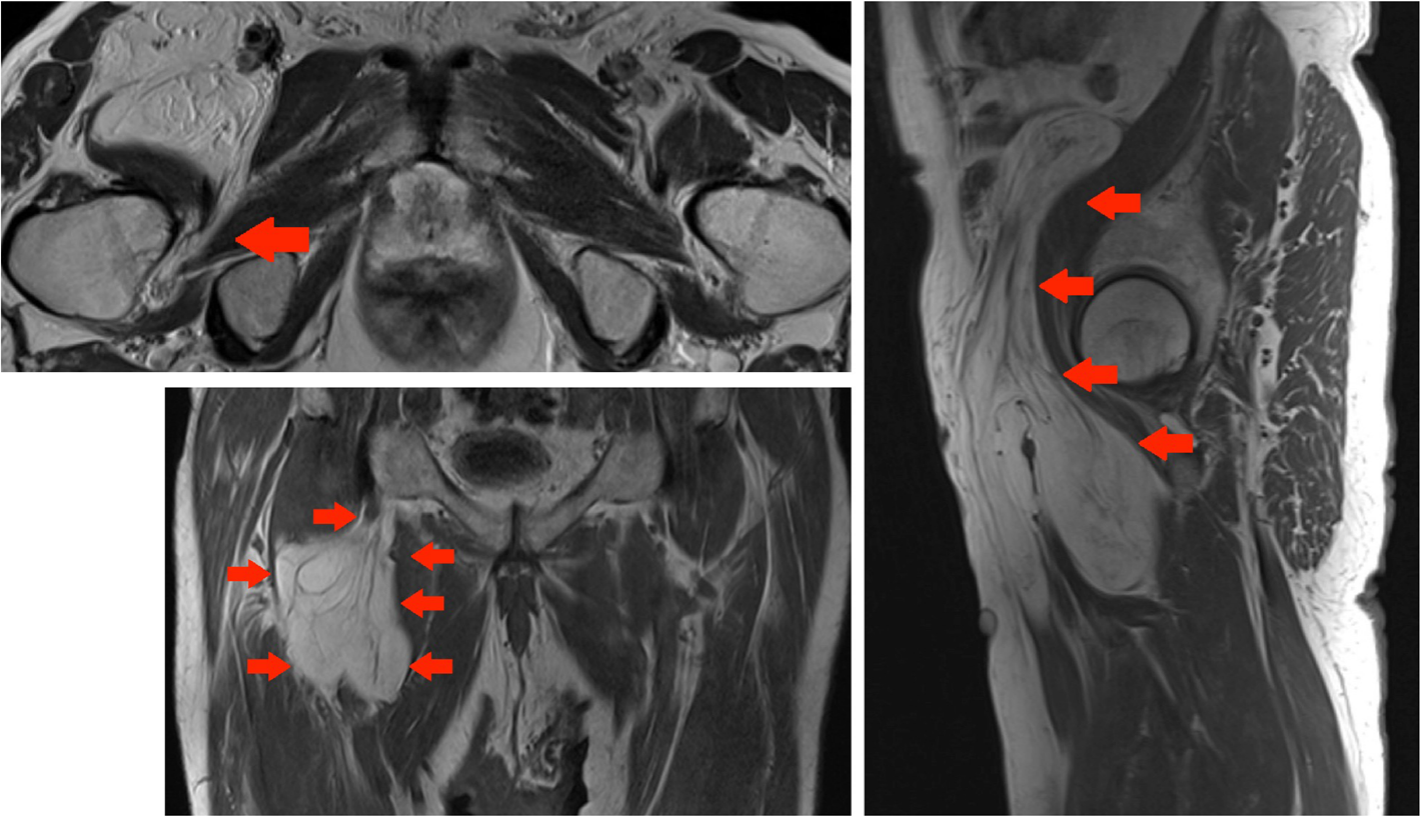
Top left panel: Axial T2-weighted MR image of the pelvis: a large lipomatous tumour arising from the right iliopsoas compartment is seen to extend to the iliopsoas insertion onto the lesser trochanter (red arrow). Bottom left panel: Coronal T1-weighted MR image of the pelvis: large lipomatous mass in the right iliopsoas compartment (red arrows). Internal septations and ‘haziness’ of the fat suggest a more aggressive lesion. Right panel: Sagittal T1-weighted image of the pelvis: Lesion of fat signal intensity continuous with the iliopsoas

A CT performed for increasing right groin pain in an 82-year-old gentleman picked up a large incidental lipomatous lesion in the right iliopsoas compartment. This was characterised on a dedicated MRI. Marginal excision was performed and cytogenetic analysis confirmed MDM2 amplification in keeping with ALT (Fig. 14).

#### Case 10 B

**Fig. 15:**
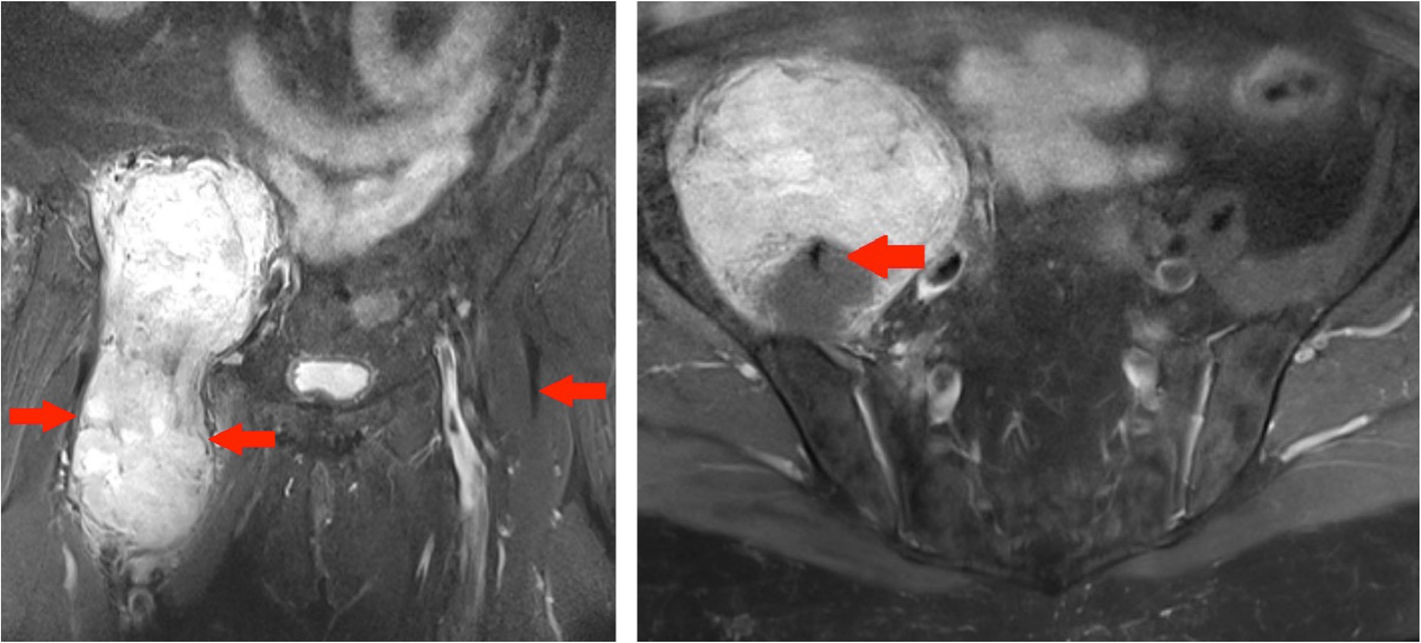
Left panel: Coronal T1 TIRM MR image of the pelvis: right-sided large heterogeneous tumour with areas of internal fat component demonstrating suppression on this fat saturated sequence. The paucity of fat components and the nodularity of the lesion suggested that radiologically this is a high grade, undifferentiated sarcoma. Right panel: Axial T1 TIRM MR image of the pelvis: the lesion is in continuity with the right iliopsoas and the red arrow outlines the iliopsoas tendon

A 72-year-old lady had complaints of right groin and buttock pain since 3 months. She had no associated weight loss. This lesion proved to be an undifferentiated high-grade pleomorphic sarcoma (Fig. 15).

#### Case 10 C

**Fig. 16:**
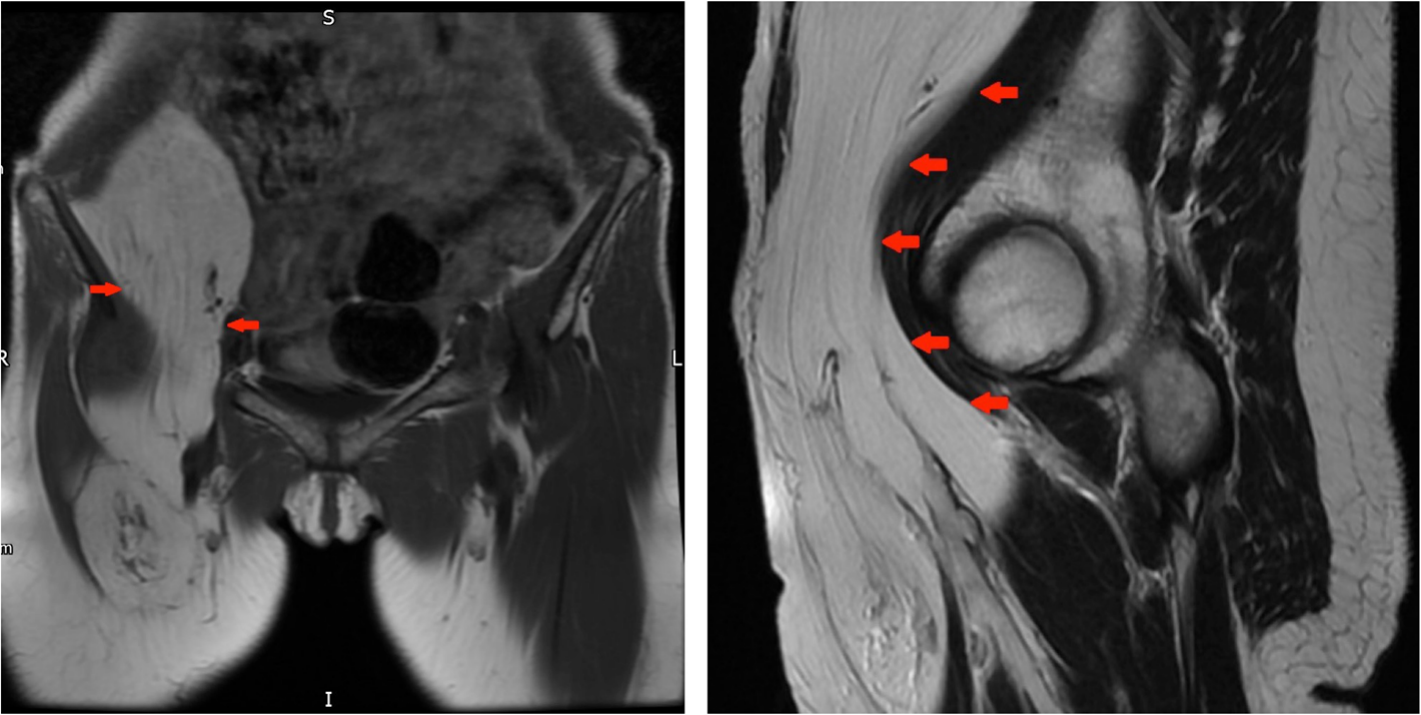
Left panel: Coronal T1-weighted MR image of the pelvis: large lipomatous tumour in the right iliopsoas compartment proved to be a well differentiated low grade liposarcoma histologically. Right panel: Sagittal T2-weighted image of the pelvis: ALT in the right iliopsoas compartment as outlined by the red arrows

A 49-year-old woman only recently noticed a lump in her right groin and anterior thigh and attributed this to a recent weight loss (2.5 stone) making the swelling more obvious (Fig. 16).

#### Case 10 D

**Fig. 17:**
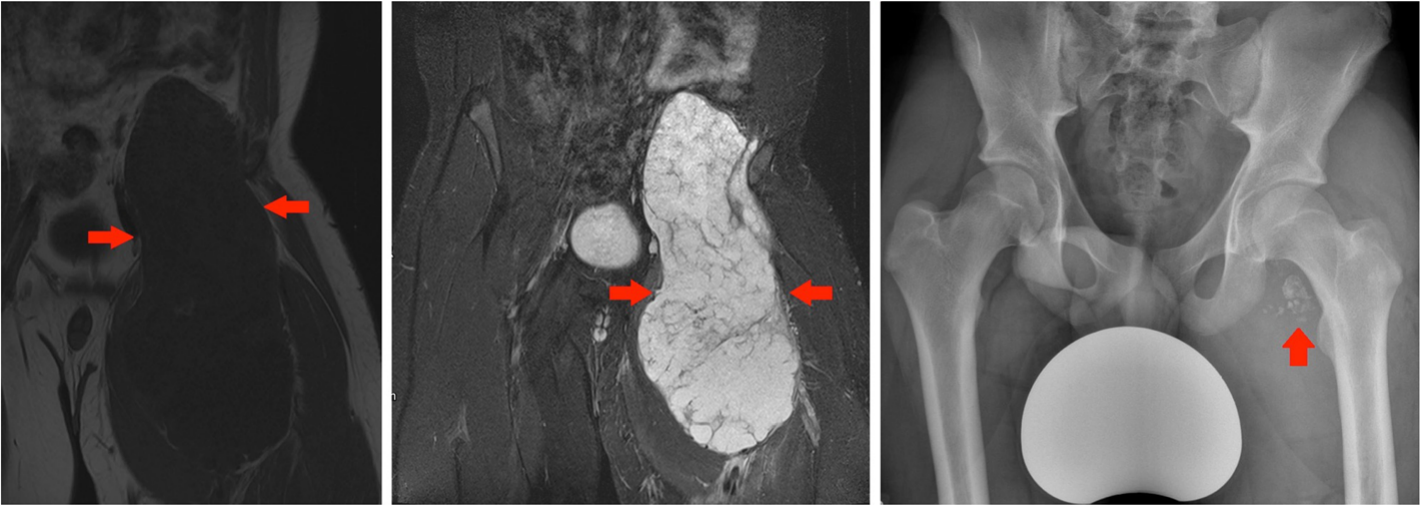
Left panel: Coronal T1-weighted image of the pelvis and upper thigh: large low signal intensity mass is seen in the left iliopsoas compartment (red arrows). This demonstrates a paucity of fat signal. Middle panel: Coronal T1 TIRM image of the pelvis and upper thigh: a high signal intensity is seen in the tumour with low signal septations and an overall aggressive appearance. Right panel: AP X-ray of the pelvis: red arrow confirms an area of intra-lesional chondroid calcifications. Biopsy confirmed a high-grade chondrosarcoma. Intra-operatively the tumour appeared to be originating from the lesser trochanter

A 16-year-old boy noticed an increase in weight and a significant enlargement of his left thigh which on clinical examination was 20 cm bigger in diameter than the right side. There was no associated pain and as he was a keen rugby player, he initially attributed these changes to a sports injury. When he finally presented to his GP, the mass in his left thigh was significantly large and he was referred for urgent imaging investigations. On MR imaging, the tumour contained small areas of fat intensity and was mostly hyperintense on fluid-sensitive fat saturated sequences, which pointed towards a (myxoid) liposarcoma. However, this case nicely illustrates that plain films are key to assess the presence of matrix calcifications in soft tissue tumours. After resection, histology was in keeping with a grade 3 periosteal chondrosarcoma (Fig. 17).

## Discussion

In this article, we have shown a wide range of lipomatous tumours in patients who presented with a palpable lesion herniating through a defect in the abdominal wall or extending in the psoas compartment.

In daily practice, this implies that however rare soft tissue sarcomas may be, the ultrasound assessment of an inguinal hernia or a quadriceps muscle strain can be the moment of detecting a sarcoma, i.e. this examination is often not performed by an MSK radiologist or in a tertiary referral centre.

We would like to summarise the take home messages for the encounter with these herniating lesions using the European Society of Musculoskeletal Radiology (ESSR) guidelines for soft tissue tumours for reference. [10]

The first diagnostic step in the characterisation of a palpable soft tissue mass is an ultrasound. Ultrasound is mainly performed to exclude clearly benign lesions such as ganglions or other cystic lesions, bursas, small subcutaneous lipomas, palmar or plantar fibromas and neurofibromas.

When assessing a superficial lipomatous lesion, it should be homogeneous and well defined, encapsulated and compressible [10]. When assessing a hernia with ultrasound, if the herniating tissue is not fully accessible, i.e. too large or the margins are not clearly visible, additional cross-sectional imaging by CT or MRI is warranted. For pelvic and retroperitoneal lesions, CT is the first step in further characterisation of the lesion.

Worrisome features for lipomatous tumours include size > 5 cm, nodularity which may represent a dedifferentiated area within the tumour, multiple septations or thickened septations within the lesion and deep location, i.e. below the superficial muscle fascia (intra- or inter-muscular, retroperitoneal). In addition, from our experience, haziness of the fat and oedema within the fatty tumour can point towards a malignant lesion.

Regarding the use of gadolinium contrast when performing an MR for the characterisation of a soft tissue tumour and in our case more specifically a lipomatous tumour, ESSR guidelines specify that static postcontrast sequences should be performed in two planes. Subtraction images and dynamic contrast enhancement may be added to this protocol but are not mandatory [10].

Comparing the T1 fat suppressed sequences performed pre- and post-intravenous gadolinium, enhancement of nodular, dedifferentiated components or septations within a lipomatous tumour can be assessed; however, the exact additional value of contrast in comparison to a non-enhanced fat-suppressed sequence remains unclear. A practical approach could be to schedule patients with lipomatous tumours as supervised MRIs. As soon as the fat-suppressed sequences have been obtained, the radiologist assesses the lesion and in case of a homogeneous fatty lesion (in comparison with the subcutaneous fat) with only thin internal septations, no contrast is needed. If however there is oedema in the lesion, or nodularity or thicker septations are seen, the protocol can be extended with post contrast series.

In addition, contrast may help in discriminating a chondroid ‘rings and arcs’ type enhancement pattern or show typical nonenhanced areas representing tumour necrosis or mucinous material within a myxoid liposarcoma [11].

We presented a case of a large retroperitoneal tumour extending into the psoas compartment, which was initially called a lipomatous tumour based on small foci of fatty signal present on the MRI. However, histology proved the lesion to be a high-grade chondrosarcoma, which should be in the differential for large lesions in the psoas compartment. The small chondroid matrix calcifications present in the soft tissue as illustrated on X-ray were the important key to the correct diagnosis. This case underlines the importance of conventional X-rays for tumour pathology.

Finally, once a lipomatous tumour has been found on imaging (whether that was an incidental finding or a finding on imaging aimed at characterisation of the lesion), referral of the patient to a specialised sarcoma treatment centre is needed before biopsy and resection. The biopsy route should be planned in agreement with the oncologic surgeon, as the biopsy tract needs to be included in the resection of the tumour to prevent needle tract seeding and local recurrence [12]. A biopsy route that does not traverse an uninvolved muscular compartment, joint or neurovascular bundle should be chosen. In addition, when biopsies are erroneously performed through large muscles such as the quadriceps or gluteal muscles, this could be a harmful procedure as the muscles would have to be included in the resection resulting in loss of function for the patient [13].

## Abbreviations

ALT: Atypical lipomatous tumour
CT: Computed tomography
ESSR: European Society of Musculoskeletal Radiology
GP: General practitioner
MRI: Magnetic resonance imaging
PET CT: Positron emission tomography computed tomography
SUV: Standardised uptake value
